# *Piriformospora* *indica* Root Colonization Triggers Local and Systemic Root Responses and Inhibits Secondary Colonization of Distal Roots

**DOI:** 10.1371/journal.pone.0069352

**Published:** 2013-07-26

**Authors:** Lorenzo Pedrotti, Martin J. Mueller, Frank Waller

**Affiliations:** Pharmaceutical Biology, Julius-von-Sachs-Institute for Biosciences, Biocenter, University of Wuerzburg, Wuerzburg, Germany; Graz University of Technology (TU Graz), Austria

## Abstract

*Piriformospora* *indica* is a basidiomycete fungus colonizing roots of a wide range of higher plants, including crop plants and the model plant *Arabidopsis thaliana*. Previous studies have shown that *P* *. indica* improves growth, and enhances systemic pathogen resistance in leaves of host plants. To investigate systemic effects within the root system, we established a hydroponic split-root cultivation system for Arabidopsis. Using quantitative real-time PCR, we show that initial *P* *. indica* colonization triggers a local, transient response of several defense-related transcripts, of which some were also induced in shoots and in distal, non-colonized roots of the same plant. Systemic effects on distal roots included the inhibition of secondary *P* *. indica* colonization. Faster and stronger induction of defense-related transcripts during secondary inoculation revealed that a *P* *. indica* pretreatment triggers root-wide priming of defense responses, which could cause the observed reduction of secondary colonization levels. Secondary *P* *. indica* colonization also induced defense responses in distant, already colonized parts of the root. Endophytic fungi therefore trigger a spatially specific response in directly colonized and in systemic root tissues of host plants.

## Introduction

The association of plant roots with beneficial fungi is extremely widespread among terrestrial plants and plays an important role in increasing host plant fitness, e.g. for nutrient uptake and resistance to abiotic and biotic stress conditions [1,2]. Fungi of several clades colonize plant roots, including arbuscular mycorrhizal fungi of the phylum *Glomeromycota* which are obligate biotrophs [3,4]. The fungal order *Sebacinales* (Basidiomycota, Agaricomycotina) shows a high morphological and physiological diversity and members have been detected worldwide [5,6]. *Piriformospora* *indica* of this order has been particularly well studied, due to its beneficial effects on growth and stress resistance of host plants and as it can be cultivated axenically [7–11].

*P* *. indica* colonizes roots of both gymnosperms and mono- and dicotyledonous angiosperms, whereas a non-host plant has not been found yet. Colonization is restricted to the rhizodermis, and the fungus does not enter the vasculature or shoot of the plant (Deshmukh et al. 2006). Colonization was not observed to reach levels negative for the host plant, and can last several months in soil-grown barley plants [11]. An initial biotrophic growth phase can be distinguished from a later phase starting 3 days post inoculation, in which cell death of colonized cells is observed [12–14]. Consequently, cell death control by the protein BAX-INHIBITOR1 is involved in controlling the level of fungal colonization, at least during the first three weeks after inoculation [12]. In addition, several plant hormone signaling pathways were shown to be required for colonization or for the induction of host responses: Ethylene signaling pathways were required for restricting the level of *P* *. indica* colonization, as well as for host plant growth induction by the root endophyte [15]. Genes involved in biosynthesis and responses to gibberellic acid and abscisic acid (ABA) were shown to be induced at specific stages of colonization [16]. The fungus is able to produce the auxin indole-acetic acid (IAA) in isolated culture [17], and influences auxin-induced genes in barley roots [16]. On the other hand, in colonized Arabidopsis roots, auxin levels were not elevated, and typical auxin-regulated marker genes were not affected, while auxin and cytokinin signaling were required for *P* *. indica* induced growth responses in this species [18,19].

Some host plant responses could be induced by diffusible signaling molecules of the fungus, which is supported by experiments showing that *P* *. indica* -induced growth promotion can also be induced by fungal extracts [19]. In addition, *P* *. indica* cells were shown to harbor *Rhizobium radiobacter* bacteria in low density, which are able to directly influence plant responses when plants were inoculated with sufficiently high amounts [20]. Bacteria-derived signaling substances, e.g. homoserine lactones, which are able to directly influence plant responses [21] could therefore also play a role in the interaction of the root endophyte with the host plant. While the timing and spatial distribution of possible fungal signaling molecules and regulation of host signaling pathways remain to be elucidated in detail, *P* *. indica* colonization induces complex, stage-specific responses of signaling pathways in the host tissue.

Contact of roots with *P* *. indica* chlamydospores triggers only a moderate response of defense-related transcripts [16]. These immediate responses to *P* *. indica* are transient and can be followed by a suppression of defense responses, which are dependent on intact jasmonic acid signaling [13]. Despite the observed repression of defense responses in colonized tissue, *P* *. indica* induces faster and stronger defense responses against the biotrophic leaf pathogen *Blumeria* *graminis* in barley, and primes defense responses against *Golovinomycesorontii* in Arabidopsis leaves [11,22]. Also, an enhanced resistance of *P* *. indica* colonized roots against the root pathogen *Fusarium* *culmorum* was observed [11].

Although systemic effects on pathogen responses were shown for the shoots of *P* *. indica* colonized plants, it is not clear how the induction of defense responses is spatially distributed in the root upon local contact with the root endophyte. We therefore tested systemic effects induced by *P* *. indica* in distal, non-colonized roots. By using a split-root system we could show that *P* *. indica* colonization is exerting systemic effects on the expression of defense-related transcripts in distal roots within one day. Furthermore, the root endophyte negatively affected secondary *P* *. indica* colonization of distal roots of the same plant. Quantitative real-time PCR revealed spatially specific induction and priming of defense-related transcripts in the root during *P* *. indica* colonization.

## Materials and Methods

### Plant and fungal material, growth conditions

**Figure 1:**
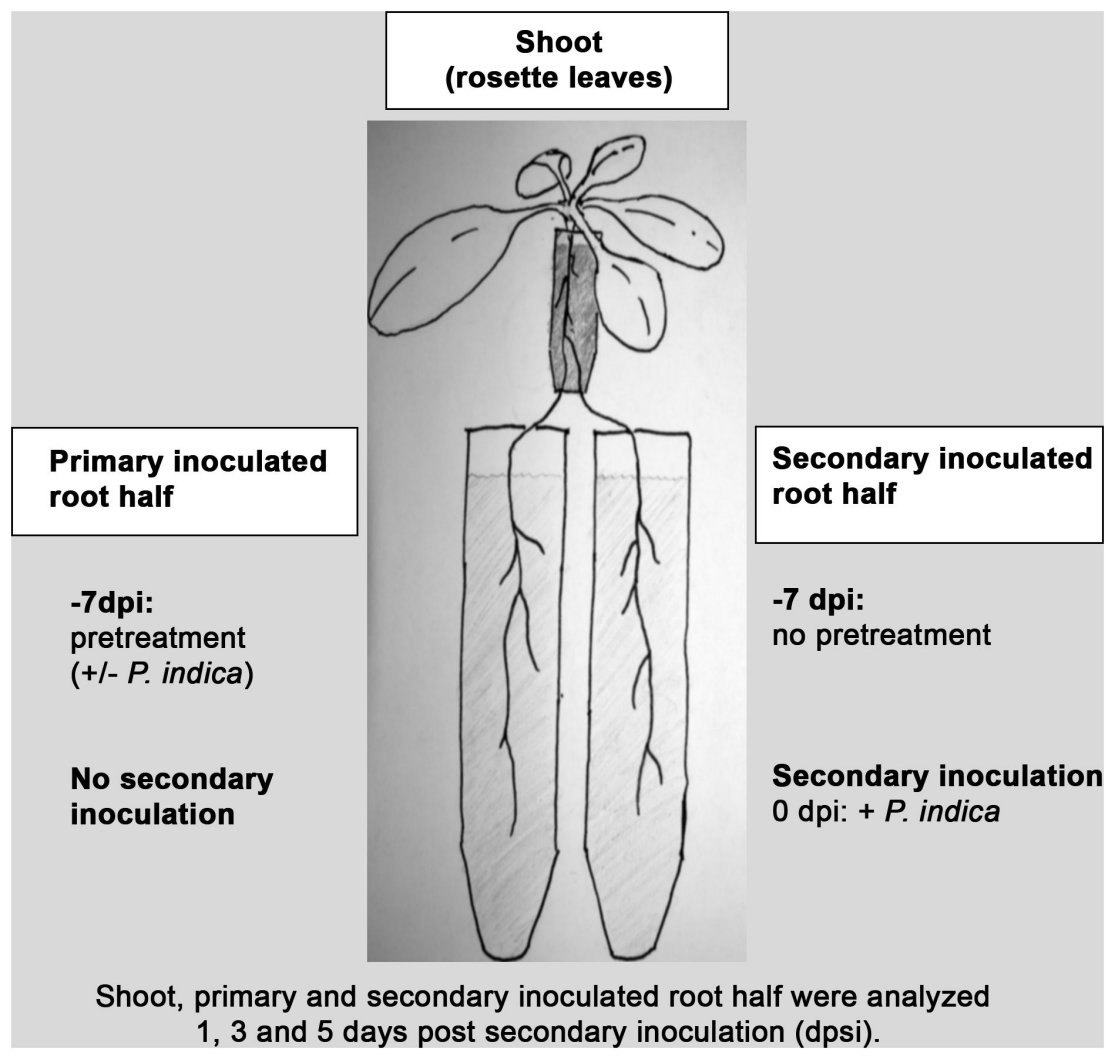
Experimental setup for pretreatment and secondary *P indica* inoculation in Arabidopsis split-root hydroponic culture. To test for effects of existing *P. indica* colonization on a subsequent contact with *P. indica* chlamydospores in distal roots, we performed pretreatment experiments with the split-root experimental system. Pretreatment was restricted to one root half (‘primary inoculated root half’), and consisted of either living or inactivated *P. indica* chlamydospores. The non-pretreated root half received a secondary inoculation with *P. indica* (‘secondary inoculated root half’). To determine the level of colonization, the two root halves were harvested separately 1, 3, 5 and 7 days post secondary inoculation (dpsi), and relative amounts of fungal DNA were determined by qPCR (Figure 4). For assessing marker gene expression, shoots and the two different root halves were harvested 1, 3 and 5 dpsi (Figure 3, Figure 5, Figure 6).

Arabidopsis thaliana ecotype Colombia (Col-0) seeds were sown in sterile 0.5 ml tubes containing 1% PhytoAgar medium prepared with Murashige and Skoog [23] Basal Salt Mixture (Duchefa Biochemie; www.duchefa-biochemie.nl). After stratification for 48 h at 4°C, plants were grown in a controlled environment growth chamber at 22/20 °C day/night cycle under short-day photoperiod (9 h light / 15 h dark). After 12 days of growth the bottom of each tube was removed and tubes were inserted into the lid of 15 ml plastic tubes containing Liquid Medium (LM), as described in [24]. Tubes were covered with aluminum foil to reduce light exposure of roots, and the medium was replaced every 7 days. Plants were grown for 23 days in hydroponic culture before tests with *P* *. indica* root inoculation were performed. To investigate root-to-root signaling, roots from hydroponic culture were split in two parts and immersed in two 15 ml tubes with LM (Figure 1). Before inoculation with *P* *. indica* , plants were grown for at least 3 days for adaptation.

*Piriformospora* *indica* was maintained on complex medium (CM), a modified Aspergillus medium [25] with agar (15g/L, Duchefa Biochemie). Chlamydospores were isolated from 3–6 week old plates and washed three times by centrifugation at 3000 g for 10 minutes using 30 ml of 10 mM MgCl_2_ solution. Spore concentration was adjusted with LM.

For microscopic investigation of root colonization, fungal cells were stained with Wheat Germ Agglutinin-Alexafluor 488 (Molecular Probes; Invitrogen, www.invitrogen.com) as described in [12]. Confocal fluorescence images and respective bright field images were recorded on a multichannel Axioplan 2 confocal microscope (Zeiss; www.zeiss.com). Host plant growth was determined by measuring the length of main roots and lateral roots manually after removing the plant from hydroponic culture and placing them on wet glass plates. Dry weight was determined after drying the plants at 60°C for 24 h.

### Split-root tests

Fungal inoculation was performed by adding 15 ml of a 5×10^5^ spore / ml *P* *. indica* spore suspension to one of the two tubes. Root and shoot samples were collected at 0, 1, 3, 5 and 7 days post inoculation (dpi) and stored at -20°C. Each sample contained materials from three different plants. For harvesting, those parts of the root not immersed in medium were removed and roots were carefully dipped into distilled water (6 times) to remove loosely associated fungal hyphae. To avoid errors due to contamination, samples of non-inoculated root halves were tested for the presence of *P* *. indica* DNA, and only *P* *. indica* -negative samples were used for analysis. To characterize the influence of a primary colonization on secondary colonization with *P* *. indica* , one tube with half of the root system was inoculated. After 7 days the other part of the roots was inoculated in the same manner and samples were collected 1, 3 and 5 days post secondary inoculation (dpsi). Control plants were not pre-inoculated and received no secondary treatment (‘control’), and ‘no pretreatment’ plants received only the secondary treatment. Inactivated spores were obtained by sonication of the spore suspension for 10 min. Inactivation of spores was confirmed by the absence of fungal structures 2 weeks after plating 0.5 ml of the spore suspension on CM medium.

### Quantitative real time PCR analysis

Total RNA was extracted using peqGOLD TriFast^TM^ (Peqlab Biotechnologie; www.peqlab.de) according to the manufacturer’s protocol. 1 μg of RNA was treated with DNase I (Fermentas; www.thermoscientificbio.com) and reverse transcribed to cDNA using RNA M-MLV reverse transcriptase (Promega; www.promega.com) following the respective protocol. Real-time PCR was performed using a Biorad Thermocycler CFX96 (BioRad; www.biorad.com). For amplification, Absolute SYBR Capillary Mix (ABGene; www.thermoscientificbio.com) was used in a final volume of 20 μl. The Cycler was programmed as follows: 95°C for 15 min followed by 37 cycles of 95°C for 15 s, 55°C for 20 s and 72°C for 20 s, and then 95°C for 10 s and 70°C for 5 s. The 2^-ΔCt^ method [26] was used to calculate the difference in expression of chosen genes using as internal standard a gene coding for a SAND family protein (At2g28390) for root samples, and of ACTIN 2/8 (At3g18780) for shoot samples, respectively. PCR primer sequences are listed in Table S1. For relative quantification of fungal colonization, genomic DNA of Arabidopsis roots and *P* *. indica* was extracted using peqGOLD TriFast^TM^ (Peqlab Biotechnologie; www.peqlab.com) according to the manufacturer’s protocol. 40 ng of DNA served as template for qPCR analysis. Fungal colonization was determined by subtracting the raw threshold cycle (Ct) values of *P* *. indica* Internal Transcribed Spacer (*PiITS*) gene from those of *AtUBQ5* (At3g62250). All experiments were repeated twice, and mean values and standard errors were determined from the three independent biological replicates. After performing a Bartlett’s test which confirmed equal variances across samples, we performed Student’s t-tests to calculate p values. Values below 0.05 were marked with an asterisk in respective figures.

## Results

### *P* *. indica* colonizes Arabidopsis roots under hydroponic culture conditions

**Figure 2:**
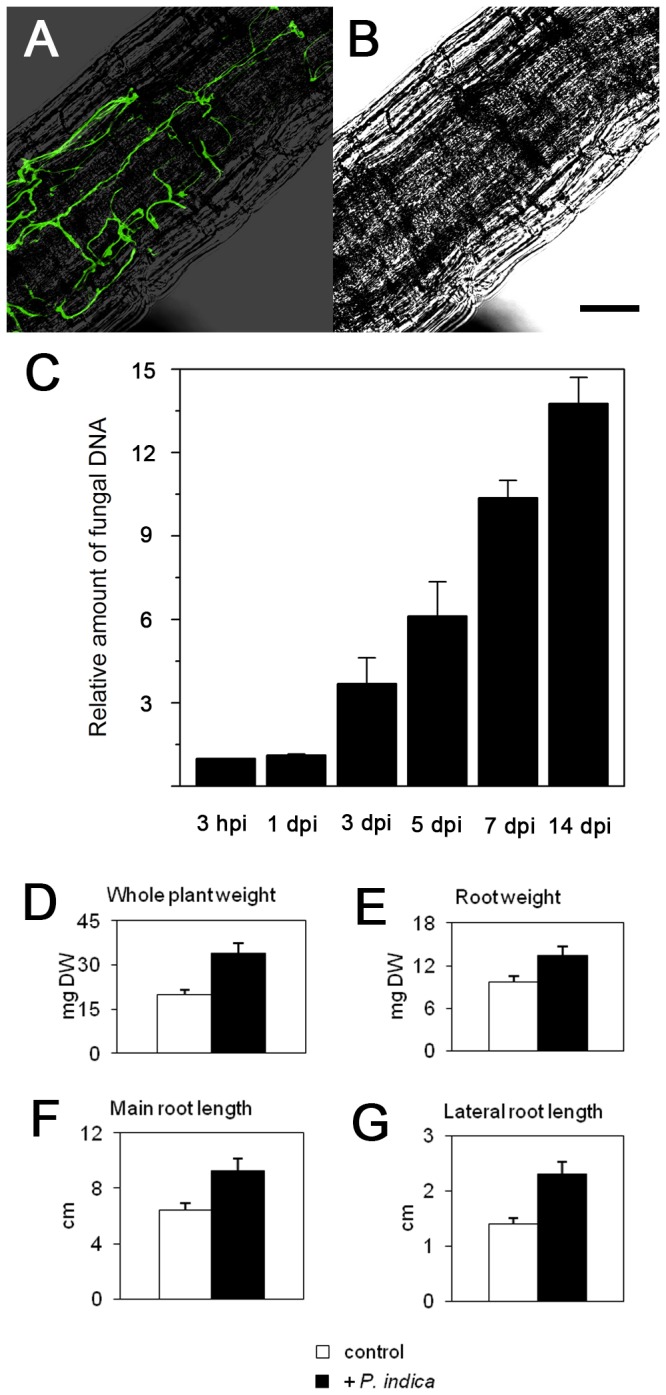
*P* *. indica* colonizes Arabidopsis roots and enhances growth under hydroponic culture conditions. A) *P. indica* colonized root grown under hydroponic culture conditions. To visualize fungal structures, Arabidopsis root sections colonized with *P. indica* were stained with WGA-AF 488 5 days after inoculation. The fluorescent image was recorded with a confocal microscope. B) The bright field image corresponding to A was merged with the fluorescent image. The scale bar represents 50 µm. C) Development of fungal colonization over time. Colonization levels were calculated as the relative amount of fungal DNA after DNA extraction of colonized root samples and subsequent quantitative PCR with *P. indica*- and Arabidopsis-specific primer pairs. Values are means of three independent experiments, with errors bars depicting standard error of the mean (SEM). D, E, F, G) Influence of *P. indica* colonization on host plants. Roots of Arabidopsis plants were inoculated with *P. indica* chlamydospores 7 days after transfer of seedlings to hydroponic culture. After four weeks, dry weight (DW) of whole plants (D) and of roots (E), as well as main root length (F) and average lateral root length (G) were determined for non-inoculated control plants (white bars) and *P. indica* inoculated plants (black bars). Values represent means of three independent experiments, each consisting of 12 plants. Error bars represent standard deviation. P-values calculated with the Student’s t-test were lower than 0.05 for all four parameters depicted in D, E, F and G.

To be able to perform split-root experiments, we used a hydroponic system for growing *Arabidopsis thaliana* (Figure 1). Fungal root colonization (Figure 2A) was similar to that observed with *A. thaliana* grown on Agar plates [22]. The development of fungal colonization was determined by quantitative PCR: Relative amounts of fungal DNA in root samples increased more than 10-fold relative to plant DNA from day 1 to day 14 after inoculation (Figure 2C).

### *P* *. indica* induces enhanced growth and changes root architecture in Arabidopsis grown under hydroponic conditions

We tested if host plant growth was affected by *P* *. indica* root colonization under hydroponic conditions. Total plant dry weight was increased by 45% in colonized as compared with non-colonized plants 3 weeks after *P* *. indica* inoculation (Figure 2D). Root dry weight was increased by 18% (Figure 2E). Main root length and length of lateral roots also significantly (p< 0.05) increased about 38 and 64 %, respectively (Figure 2F, 2G). When roots of further developed plants (5 weeks after sawing) were inoculated, no significant changes in growth could be observed within the following 14 days of plant growth, except that length of lateral roots was negatively affected (Figure S1). The latter conditions were used for all subsequent split-root experiments to minimize the influence of developmental differences on gene expression patterns.

### *P* *. indica* colonized roots influence gene expression in distal, non-colonized roots

**Figure 3:**
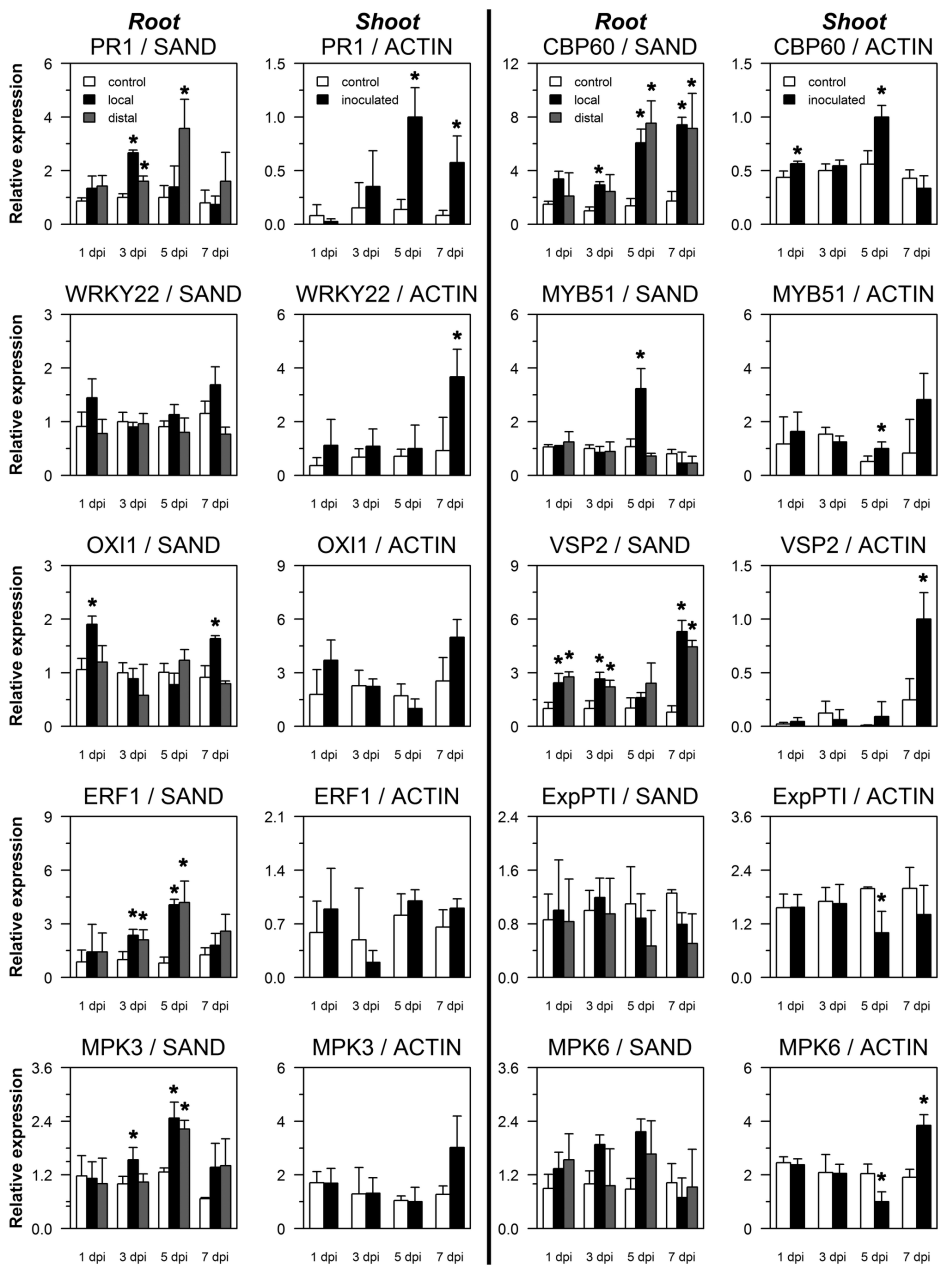
*P. indica* colonization influences gene expression in distal, non-colonized roots. Expression of defense-related genes *PR1*, *CBP60*, *WRKY22*, *MYB51*, *OXI1*, *VSP2*, *ERF1*, *ExpPTI*, *MPK3 and MPK6* in roots and shoots of hydroponically grown split-root plants. Shown are expression levels relative to the constitutive *SAND* transcript in *P. indica* colonized root halves (‘local’), non-colonized root halves (‘distal’) and in roots of non-colonized plants (‘control’). For shoots, expression levels are relative to the *Actin 2/8* transcripts for *P. indica* inoculated (‘inoculated’) and non-inoculated plants (‘control’). Values are means of three independent experiments, with errors bars depicting standard error of the mean (SEM). Asterisks mark those values different from respective control treatments with p values lower than 0.05 calculated in Student’s t-tests.

Transcripts of marker genes indicative of different signaling pathways were tested for their expression in colonized, ‘local’ roots, non-colonized, ‘distal’ roots, and in shoots (rosette leaves) 1, 3, 5 and 7 days post inoculation (dpi). SA-regulated *PR1* was induced about 2.5-fold in local roots 3 dpi, whereas a substantial induction in distal roots was observed 5 dpi (Figure 3). Both inductions were transient, with no significant differences to control plants at 1 dpi and 7 dpi. In the shoot, a similar kinetic was observed for *PR1* as in distal roots, with highest induction 5 dpi. SA-responsive *CBP60* (*Calmodulin-binding protein 60-like G*) was elevated 3 and 5 dpi in both local and distal roots, while in the shoot higher transcript levels were observed 5 dpi. JA-regulated *VSP2* levels were significantly higher in both local and distal roots 1, 3 and 7 dpi. In the shoot higher *VSP2* levels were detected 7 dpi. Ethylene-responsive *ERF1* transcripts were elevated both in local and distal roots 3 and 5 dpi, but not in the shoot. GA-regulated *ExpPT1* (*Phosphatidylinositol N-acetylglucosaminyltransferase subunit P-related*), and *WRKY22*, a marker for MAMP-triggered immunity, showed no significant induction. *OXI1* (*Oxidative signal inducible1*, also known as *AGC kinase family protein AGC2-1*), indicative of oxidative stress, was weakly induced locally 1 and 7 dpi, and *MYB51*, indicative for glucosinolate production, was induced only locally at 5 dpi. Mitogen-activated protein kinase 3 (MPK3) was upregulated locally 3 dpi and both locally and systemically 5 and 7 dpi. A range of mainly SA and JA-regulated transcripts was therefore induced by *P* *. indica* at specific time points not only in inoculated, but also in distal parts of the root, and in some cases also in the leaves.

### *P* *. indica* colonized roots inhibit subsequent *P* *. indica* colonization of distal roots

**Figure 4:**
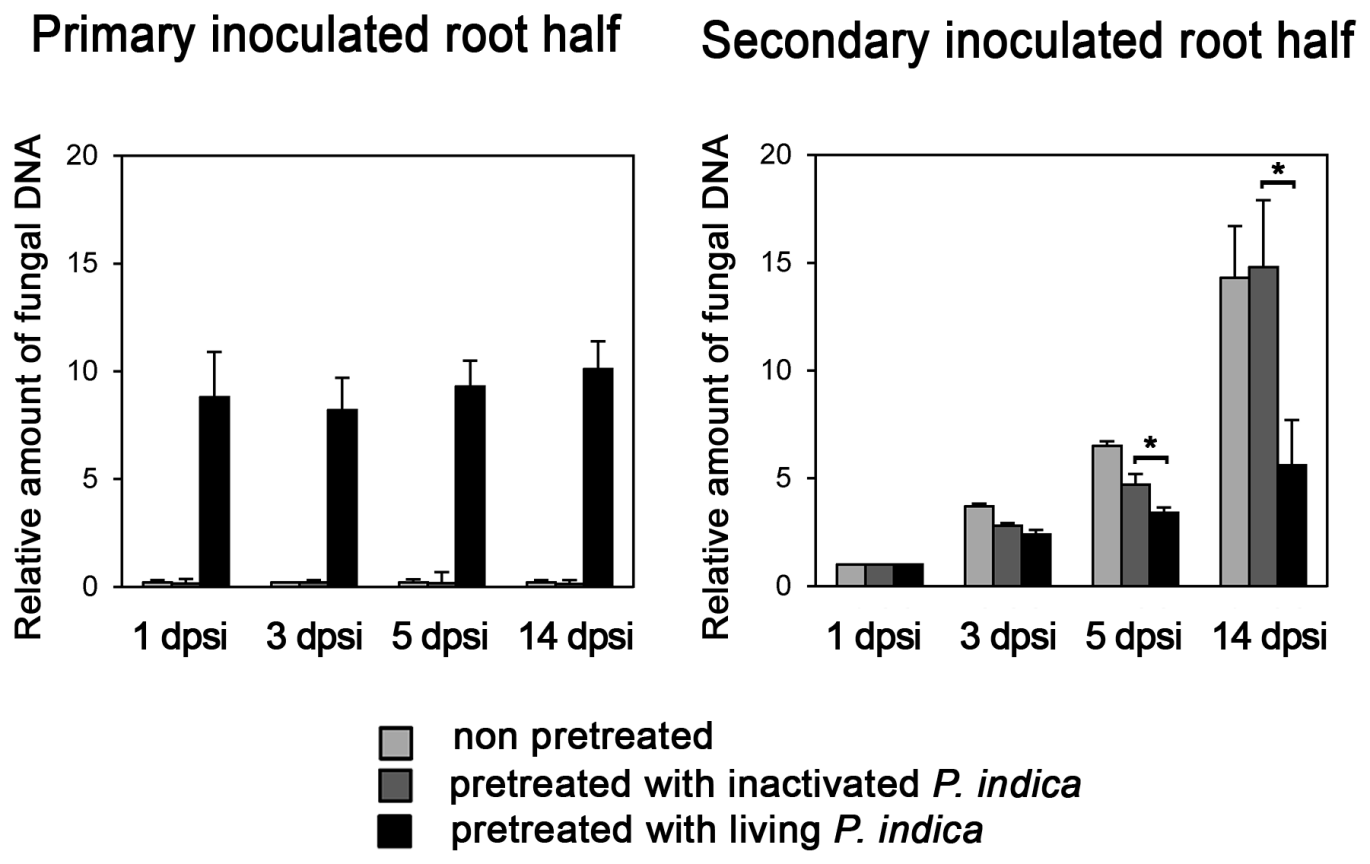
*P. indica* colonization inhibits subsequent *P. indica* colonization of distal roots. Plants grown in the split-root hydroponic system were pre-inoculated with living or inactivated *P. indica* chlamydospores on one root half (Figure 1). After 7 days, the other, non-inoculated root half was subjected to a secondary root inoculation with *P. indica.* Both the secondary inoculated root half, and the pretreated root half (‘primary inoculated root half’) were analyzed 1, 3, 5 and 14 days post secondary inoculation (dpsi). Colonization levels were calculated as relative amounts of fungal DNA after DNA extraction of root samples and subsequent quantitative PCR with *P. indica*- and Arabidopsis-specific primer pairs. Values are means of three independent experiments, with errors bars depicting standard error of the mean (SEM). Asterisks mark those values of secondary inoculated roots with p values lower than 0.05 calculated in pairwise Student’s t-tests between pretreatment with inactivated and with living *P. indica.*

Previous studies showed that *P* *. indica* is colonizing roots of the host plant only to a certain degree. To study the effect of *P* *. indica* colonized roots on the degree of a secondary colonization, we set up a split root experiment in which one root half was pre-inoculated with *P* *. indica* (primary inoculated root half, Figure 1). 7 days after this pretreatment, the other root half was inoculated with *P* *. indica* (secondary inoculated root half). 1, 3, 5 and 14 days post secondary inoculation (dpsi), the relative amount of fungus was separately determined in both root halves by quantitative real-time PCR (qPCR). Pretreatment with inactivated spores reduced secondary fungal growth only transiently by 35% (3 dpsi) and 40% (5 dpsi), whereas no difference to non-pretreated controls could be observed 14 dpsi (Figure 4). In contrast, pre-treatment with living *P* *. indica* spores reduced the relative fungal amount in secondary inoculated root halves from 3 dpsi, with a pronounced 55% reduction, as compared with non-pretreated roots, at 14 dpsi. Therefore, *P* *. indica* had a negative, systemic effect on colonization levels of subsequently colonized roots. In addition, relative amounts of *P* *. indica* in the pretreated root half were also negatively affected by the secondary inoculation, as levels in the pretreated root half did not increase between 1 and 14 dpsi.

### Established *P* *. indica* colonization changes local and distal root responses to secondary *P* *. indica* colonization

As a first step to understand differences in secondary *P* *. indica* colonization levels, we pre-inoculated one root half with either living or inactivated *P* *. indica* chlamydospores. Seven days later, we performed a secondary inoculation of the previously non-inoculated root half with *P* *. indica* . 1, 3 and 5 days after this secondary inoculation (days post secondary inoculation, dpsi) we determined marker gene expression in the secondary inoculated root part, in the pretreated root part and in the shoot.

**Figure 5:**
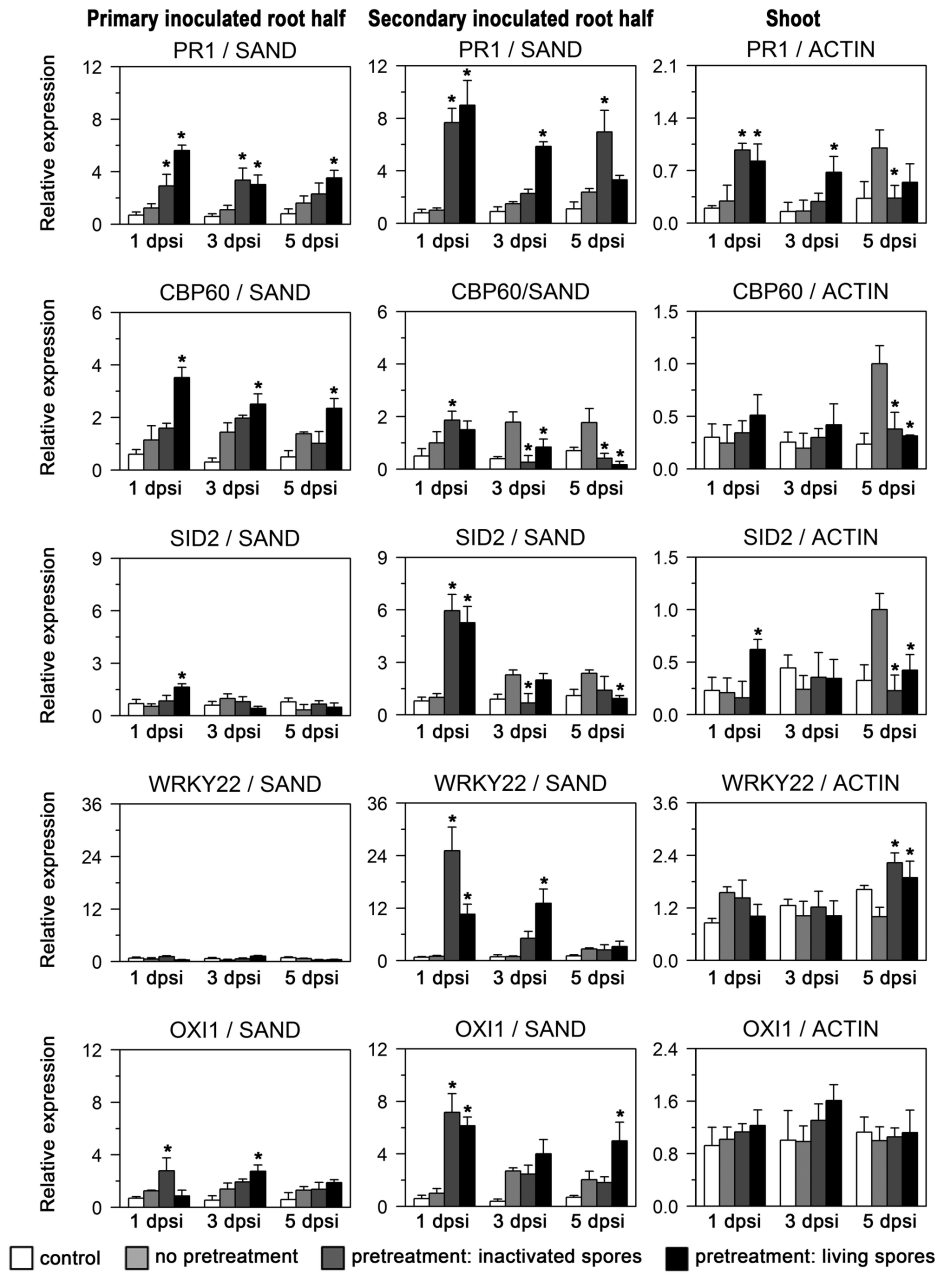
*P. indica* colonized root halves influence marker gene expression after secondary *P. indica* inoculation. Expression of defense-related genes *PR1*, *CBP60*, *SID2*, *WRKY22*, *OXI1* in roots and shoots of hydroponically grown split-root plants pre-inoculated with *P. indica*. For this experiment, plants were analyzed 1, 3 and 5 days post secondary root inoculation (dpsi) with *P. indica*, which was performed 7 days after the initial pretreatment. Shown are expression values for four different treatments: Control plants were not pretreated and received no secondary inoculation (‘control’). Plants with no pretreatment receiving a secondary *P. indica* inoculation (‘no pretreatment’), and plants with one root half pre-inoculated with either living (‘pretreatment: living spores’) or with inactivated *P. indica* chlamydospores (‘pretreatment: inactivated spores’) receiving a secondary inoculation of the non-pretreated root half with *P. indica*. Secondary inoculated root halves, primary inoculated root halves (pretreated root halves), and shoots were harvested separately at indicated time points post secondary inoculation. Expression values are relative to the constitutive *SAND* transcript for roots and to *Actin 2/8* transcripts for shoots. Values are means of three independent experiments, with errors bars depicting standard error of the mean (SEM). Asterisks mark values of pretreated plants which had p values lower than 0.05 (Student’s t-test) when compared with respective values of non-pretreated plants.

**Figure 6:**
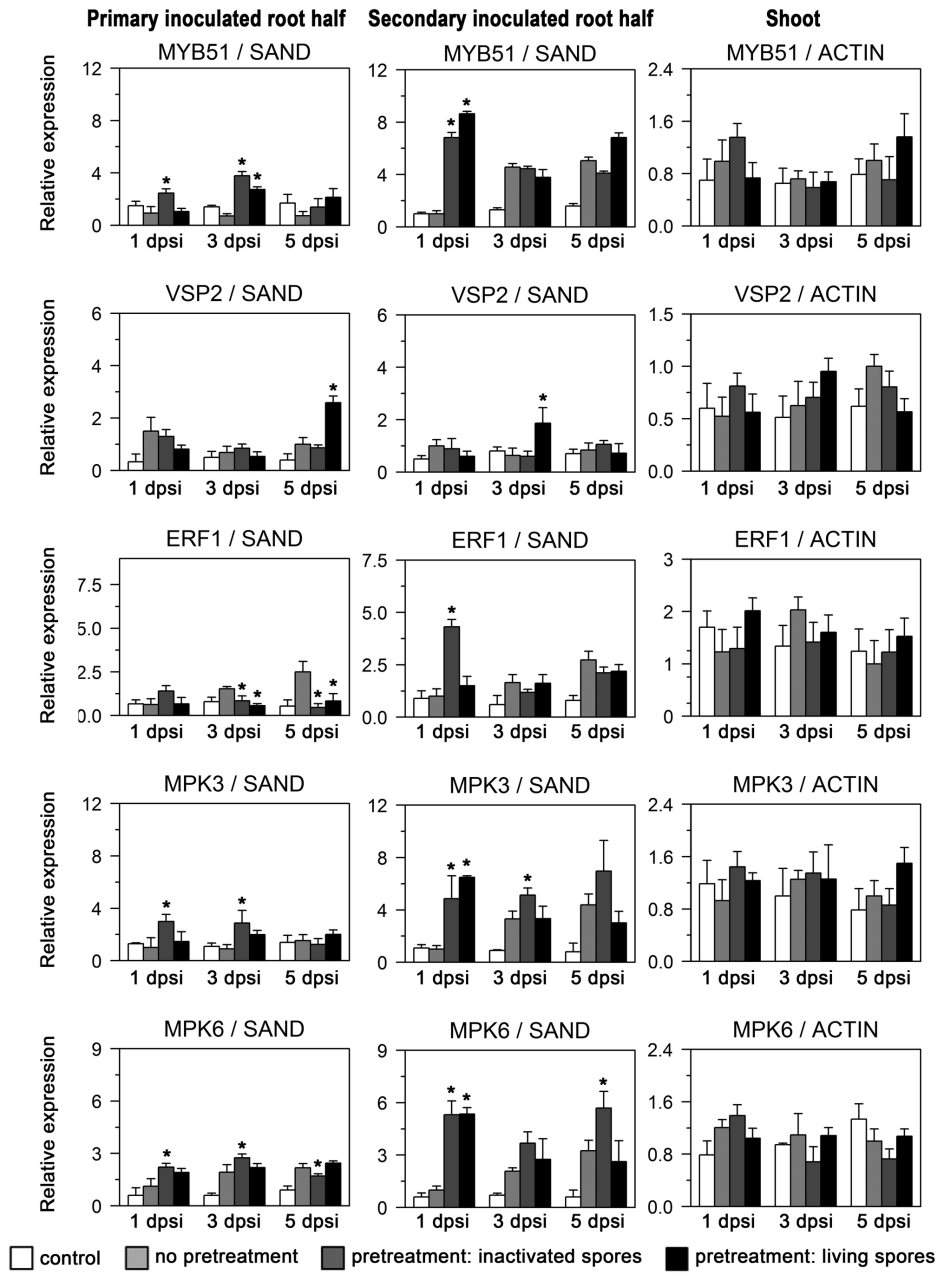
*P. indica* colonized root halves influence marker gene expression after secondary *P. indica* inoculation. Expression of defense-related genes *MYB51*, *VSP2*, *ERF1*, *MPK3* and *MPK6* in roots and shoots of hydroponically grown split-root plants pre-inoculated with *P. indica.* For this experiment, plants were analyzed 1, 3 and 5 days post secondary root inoculation (dpsi) with *P. indica,* which was performed 7 days after the initial pretreatment. Shown are expression values for four different treatments: Control plants were not pretreated and received no secondary inoculation (‘control’). Plants with no pretreatment receiving a secondary *P. indica* inoculation (‘no pretreatment’), and plants with one root half pre-inoculated with either living (‘pretreatment: living spores’) or with inactivated *P. indica* chlamydospores (‘pretreatment: inactivated spores’) receiving a secondary inoculation of the non-pretreated root half with *P. indica.* Secondary inoculated root halves, primary inoculated root halves (pretreated root halves), and shoots were harvested separately at indicated time points post secondary inoculation. Expression values are relative to the constitutive *SAND* transcript for roots and to *Actin 2/8* transcripts for shoots. Values are means of three independent experiments, with errors bars depicting standard error of the mean (SEM). Asterisks mark values of pretreated plants which had p values lower than 0.05 (Student’s t-test) when compared with respective values of non-pretreated plants.

Compared to non-pretreated plants, the local root response to secondary *P* *. indica* colonization was faster and stronger at 1 dpsi for *PR1*, *CBP60*, *SID2*, *WRKY22*, *OXI1*, *MYB51*, *MPK3* and *MPK6* (Figure 5, Figure 6). The pretreated root half was also influenced: *PR1* was induced in these roots distal to the secondary treatment with living chlamydospores about 4-fold 1 dpsi, a significantly higher and earlier induction as observed for distal roots of non-pretreated plants. Inactivated fungal spores also induced higher *PR1* levels as compared to non-pretreated samples, but to a lesser degree (at least 1 and 5 dpsi) than pretreatment with living spores. Similar to *PR1*, *CBP60* was elevated in the pretreated root half 1, 3 and 5 dpsi. In the secondary inoculated root itself, a weaker induction of CBP60 was detected for pretreatment with living spores at the three time points. *SID2* induction was weak in pretreated root halves distal to the secondary inoculation, and strong in the secondary inoculated root half 1 dpsi for both pretreatments with living and inactivated spores.

*WRKY22*, a marker for MAMP-triggered immunity, showed strong induction by living and inactivated spores only in secondary inoculated root halves. *OXI1*, indicative of oxidative stress, was induced in the pretreated root half weaker (1 dpsi with inactivated spores and 3 dpsi with living spores) than in the secondary inoculated root half. JA-regulated *VSP2* was expressed transiently higher in secondary inoculated root halves 3 dpsi and in pretreated root halves only 5 dpsi when pretreatment consisted of living spores. Both *MPK3* and *MPK6* were induced in pretreated root halves by inactivated spores, whereas the secondary inoculated roots displayed higher transcript levels with both autoclaved and living chlamydospores 1 dpsi. Taken together, secondary inoculation with *P* *. indica* led to a primed response of almost all tested defense-related transcripts in roots distal to the primary inoculated roots. Also, the primary inoculated roots itself reacted with a significant induction of some defense-related transcripts to the secondary inoculation of the other root half.

## Discussion

To elucidate if *P* *. indica* influences distant, non-colonized parts of the root we established a hydroponic split-root system. We confirmed microscopically that the fungus colonized Arabidopsis roots with a colonization pattern similar to that of plants grown on Agar plates [22], and detected a relative increase of fungal DNA levels in colonized roots over time (Figure 2). Fresh weight of plants increased after 3 weeks, compared to non-colonized plants, when young plants were inoculated with *P* *. indica* (Figure 2). Hydroponic conditions -with abundant supply of water and mineral nutrients-therefore allowed fungal colonization, which resulted in enhanced host plant growth. This is in line with previous results showing positive effects of *P* *. indica* on soil-grown barley plants independent of limiting conditions for water, nitrogen or phosphate [27].

In previous studies it was shown that *P* *. indica* root colonization has systemic effects on the shoot, e.g. enhanced systemic resistance of Arabidopsis leaves against *Golovinomycesorontii* [22]. While priming of jasmonic acid responsive genes in the leaf was shown, the nature of the systemic signal produced or induced by *P* *. indica* in the roots, as well as systemic effects within the root system remained elusive [28]. We show here that *P* *. indica* systemically influences marker gene expression in distant, non-colonized parts of the root. We could distinguish specific patterns of local and systemically influenced transcripts: SA-regulated *CBP60*, ET-regulated *ERF1*, and JA-regulated *VSP2* were upregulated at the same time points both in local and in distal, non-colonized roots (Figure 3). On the other hand, induction of *PR1* was highest 3 dpi in local roots, and 5 dpi in distal roots, similar in timing to the induction in the shoot 5 dpi. Whereas a more detailed kinetic would be needed to exactly calculate the speed of gene induction, our results suggest that there are fast and slow signals (or combinations of signals) leading to specific, *P* *. indica* induced gene expression in distal root tissues. As the fast induction of defense-related transcripts could be also triggered by inactivated fungal material (Figure 5, Figure 6), they can be characterized as MAMP-triggered responses. Interestingly, Felle et al. [29] have shown fast responses of apoplastic root pH upon *P* *. indica* inoculation, which were different for living and inactivated chlamydospores. Other fast signaling mechanisms induced by the root endophyte were increased cytosolic Ca^2+^ concentrations and MAPK phosphorylation [30]. It remains to be analyzed in further detail which other long-distance signals are required for the observed responses in distal roots. It is possible that signaling processes like reactive oxygen species produced by NADPH oxidases [31], or the fast responses recently described for jasmonates [32] play a role, especially since the *P* *. indica* -induced suppression of some defense responses was described to be JA-dependent [13].

Pre-inoculation of one root half with *P* *. indica* had a significant effect on the response of the previously non-colonized root half to a secondary inoculation: Compared with a first-time encounter with *P* *. indica* (Figure 3), *PR1*, *CBP60*, *SID2*, *WRKY22*, *OXI1*, *MYB51*, *MPK3* and *MPK6* were induced faster and stronger 1 dpsi (Figure 5, Figure 6). At this 1-day time point of the secondary inoculation, transient induction from the pretreatment, e.g. of *PR1*, was not detectable anymore, confirming the previously observed return to basal levels 7 dpi (Figure 3). The enhanced induction 1 dpsi can thus be characterized as a primed response of roots distal to *P* *. indica* pre-inoculation.

Priming, the acquired ability of cells to react to a stimulus, e.g. a pathogen, faster and / or stronger [33], can be triggered in plants by MAMPs, effectors, wounding and other factors. *P* *. indica* root colonization was shown to prime the response of Arabidopsis leaves to the biotrophic pathogen *Golovinomycesorontii* about 3 weeks after inoculation of roots with *P* *. indica* [22]. In this study, we show that also root tissue can be primed by the endophytic fungus. As inactivated *P* *. indica* chlamydospores could also trigger priming 7 dpi, molecular patterns of the fungus, but not necessarily living chlamydospores, are sufficient for initiating this process. Also, it is possible that activity of *P* *. indica* preparations is –at least in part-due to bacteria-derived molecules, as the fungus is associated with *Rhizobium radiobacter* [20]. An important priming mechanism is a higher abundance of MPK3 and MPK6, which, upon secondary contact with a pathogen, enables a fast MAP kinase-mediated signal amplification [34]. We detected that *P* *. indica* is triggering higher *MPK3* transcript levels in local and systemic roots (Figure 3), indicating that higher MPK3 levels could be the basis of root priming by the endophyte.

Several studies noted that *P* *. indica* does not ‘overgrow’ the host plant (e.g. [12]), indicating that mechanisms exist which lead to stable levels of colonization. Our experiments with split-root plants show that secondary colonization with *P* *. indica* was inhibited when the other half of the root system was previously inoculated with the fungus (Figure 4). Inactivated *P* *. indica* chlamydospores initially also reduced secondary colonization of systemic roots, but not in the long run (14 dpsi). At this time point, previous distal colonization with the endophyte reduced secondary colonization by about 50%. Therefore, only living *P* *. indica* colonizing the root has a long-lasting, restricting effect on secondary colonization of systemic roots. The effect of autoclaved fungus could be assigned to the presence of MAMPs in this treatment, an effect which can be also accomplished by flg22 treatment [13]. Mechanistically, primed defense responses, as observed for secondary *P* *. indica* colonization, could explain the reduced colonization. Primed defense responses were also observed for pretreated root halves upon distal, secondary inoculation (compare *PR1*; Figure 5). Therefore, also root tissue already colonized by *P* *. indica* is reacting stronger to a secondary inoculation of distal root tissues. Secondary *P* *. indica* contact therefore leads not only to reduced secondary colonization due to primed defense responses, but also negatively affects colonization of previously colonized roots. Of course, other mechanisms, for example, changes in assimilate allocation, as observed for AM fungi [35], are also relevant for the degree of fungal colonization.

*P* *. indica* colonization was shown to be able to suppress MAMP-triggered responses, measured as root oxidative bursts in response to different elicitors [13]. While we did not test secondary inoculation of colonized roots with inactivated *P* *. indica* , which would be more comparable to experiments by [13], but only tested secondary inoculation of systemic, distal roots, we did not observe suppression on the level of defense-related marker gene expression (Figure 5, Figure 6). The only *P* *. indica* -dependent suppression was observed transiently in distal roots for *VSP2*, at 1 day after secondary inoculation. Initial colonization by *P* *. indica* induced *VSP2* about 2-fold 1 and 3 dpi (Figure 3). In contrast, using 3-week-old Arabidopsis plants grown on agar plates, Jacobs et al. (2011) [13] reported a suppression of *VSP2* relative to *UBQ5* 7 days after *P* *. indica* inoculation compared to the mock treatment, which increased from 0 to 7 dpi about 3-fold. We did not observe such a relative repression of *VSP2*, which might be due to experimental differences and use of different transcripts for calculating relative transcript abundance. Besides *VSP2*, expression patterns of almost all other genes used both in [13] and in this study were markedly similar, e.g. local induction of *CBP60* 3 and 7 dpi, or fast induction of *OXI1* 1 dpi, indicating a robust, conserved timing of Arabidopsis root colonization and host plant responses under different experimental conditions, including hydroponic culture. Using the split-root system, we show that the host plant reaction to *P* *. indica* differs between inactivated and living chlamydospores and is spatially specific, with clear differences between a one-time inoculation with proceeding colonization, and multiple contacts of colonized and non-colonized root areas with the fungus. For plants growing under natural conditions, we expect that -as shown in the split-root experiments-multiple contacts with root endophytes of the order *Sebacinales*, as well as other microorganisms lead to root-wide induction, and not suppression, of defense responses.

Taken together, we show that *P* *. indica* colonizes and exerts beneficial effects on host plant growth in a hydroponic system. We show that *P* *. indica* is able to trigger responses in systemic, non-colonized root tissues several centimeters apart. Marker gene induction was fast within 1 day for several transcripts, and was shifted from 3 dpi locally to 5 dpi systemically for *PR1*. Earlier observations indicated that the level of colonization with the root endophyte is controlled by the host plant [12]. We quantified the negative effect of *P* *. indica* on subsequent colonization of distal roots. The observed 50% reduction could be due to priming of defense responses, which was detected for several defense-related transcripts after secondary *P* *. indica* colonization. The hydroponic split root system offers the possibility to detect spatial differences in gene expression, but also metabolites and signals important for systemic responses and for controlling the level of colonization, an important aspect of mutualistic plant–fungus relationships.

## Supporting Information

Figure S1

*P* *. indica* short-term effects on root fresh weight and root architecture in Arabidopsis grown under hydroponic conditions. Roots of Arabidopsis plants were inoculated with *P* *. indica* four weeks after transfer of seedlings to hydroponic culture. After one week and after two weeks, whole plant weight (dry weight, DW), Root weight (DW), main root length and lateral root length was determined for non-inoculated control plants and *P* *. indica* inoculated plants. Values represent means of three independent experiments, each consisting of 12 plants. Error bars represent standard deviation.
(TIF)

Table S1 List of primer pairs used for quantitative PCR. (PDF)
