# Supplementary material for: Piriformospora indica Root Colonization Triggers Local and Systemic Root Responses and Inhibits Secondary Colonization of Distal Roots
Source: PLoS One. 2013 Jul 26;8(7):e69352. doi: 10.1371/journal.pone.0069352 (PMC3724858; doi:10.1371/journal.pone.0069352)
Supplement: Table S1 — (PDF) [file pone.0069352.s002.pdf]

Supplementary Table 1. List of primer pairs used for quantitative PCR

Gene	Gene ID	Abbreviation	PCR primer pair	Defence signaling pathway	Reference (usage as marker gene)
**Pathogenesis related gene 1**	At2g14610	PR1	fw 5'-TCTAAGGGTTCACAACCAG-3'<br>rev 5'-CGCAGCGTAGTTGTAGTTA-3'	**Salicylic acid**	[1]
**Vegetative storage protein 2**	At5g24770	VSP2	fw 5'-TTGAACCCATCATACTCAG-3'<br>rev 5'-CCATTGATCTCCGATATTG-3'	**Jasmonic acid**	[2]
**Ethylene response factor 1**	At3g23240	ERF1	fw 5'-GCCGTCTTCTTCGTCTTCC-3'<br>rev 5'-TTGACCGGAACAGAATCCA-3'	**Ethylene**	[1]
**Calmodulin binding protein 60-like**	At5g26920	CBP60	fw 5'-AAGAAGAATTGTCCGAGAGGAG-3'<br>rev 5'-GGCGAGTTTATGAAGCACAG-3'	**MAMP-triggered immunity / Salicylic acid**	[3][2]
**Salicylic acid induction deficient 2**	At1g74710	SID2	fw 5'-TCCGTGACCTTGATCCTTTC-3'<br>rev 5'-ACAGCGATCTTGCCATTAGG-3'	**Salicylic acid**	[3][2]
**WRKY transcription factor 22**	At4g01250	WRKY22	fw 5'-ATCTCCGACGACCACTATTG-3'<br>rev 5'-TCATCGCTAACCACCGTATC-3'	**MAMP-triggered immunity**	[2]
**Oxidative signal inducible 1**	At3g25250	OXI1	fw 5'-TCATCTACATTGGCCGTGTC-3'<br>rev 5'-CGTCGCTCCATACAACATCT-3'	**Oxidative stress**	[4][2]
**Myb domain protein 51**	At1g18570	MYB51	fw 5'-ACCAACCTCGAATCTTCTCTG-3'<br>rev 5'-TTTCAACACAAGACTCCTCCA-3'	**Glucosinolate biosynthesis**	[5][2]
**Mitogen activated protein kinase 3**	At3g45640	MPK3	fw 5'-TGACGTTTGACCCCAACAGA-3<br>rev 5'-CTGTTCCTCATCCAGAGGCTG-3'	**Priming**	[6][7]
**Mitogen activated protein kinase 6**	At2g43790	MPK6	fw 5'-CCGACAGTGCATCCTTTAGCT-3'<br>rev 5'-TGGGCCAATGCGTCTAAAAC-3'	**Priming**	[6][7]
**Phosphatidylinositol N-acetyglucosaminlytransferase subunit P-like protein**	At2g45900	ExpPT1	fw 5'-GGATTTCATTCGTCAAACCT-3'<br>rev 5'-CAACCAATATCAAAGCGGAG-3'	**Gibberellic acid**	[8][2]
**Ubiquitin 5**	At3g62250	UBQ5	fw 5'-CCAAGCCGAAGAAGATCAAG-3'<br>rev 5'-ACTCCTTCCTCAAACGCTGA-3'	***Reference gene***	[1]
**Actin 2/8**	At3g18780	ACTIN	fw 5'-GGTGATGGTGTGTCT-3'<br>rev 5'-ACTGAGCACAATGTTAC-3'	***Reference gene***	[9]
**SAND family protein**	At2g28390	SAND	fw 5'-AACTCTATGCAGCATT-3'<br>rev 5'-GGTGGTACTAGCACAA-3'	***Reference gene***	[9]
***P. indica* intragenic transcribed spacer**	-	*Pi* ITS	fw 5'-CAACACATGTGCACGTCGAT-3'<br>rev 5'-CCAATGTGCATTCAGAACGA-3'	***Reference gene***	[10]

References for Supplementary Table 1:

1. Stein E, Molitor A, Kogel KH, Waller F (2008) Systemic resistance in Arabidopsis conferred by the mycorrhizal fungus Piriformospora indica requires jasmonic acid signaling and the cytoplasmic function of NPR1. Plant & Cell Physiology 49: 1747-1751.
2. Jacobs S, Zechmann B, Molitor A, Trujillo M, Petutschnig E, et al. (2011) Broad-spectrum suppression of innate immunity is required for colonization of Arabidopsis roots by the fungus Piriformospora indica. Plant Physiology 156: 726-740.
3. Wang L, Tsuda K, Sato M, Cohen JD, Katagiri F, et al. (2009) Arabidopsis CaM binding protein CBP60g contributes to MAMP-induced SA accumulation and is involved in disease resistance against Pseudomonas syringae. PLoS Pathogens 5: e1000301.
4. Rentel MC, Lecourieux D, Ouaked F, Usher SL, Petersen L, et al. (2004) OXI1 kinase is necessary for oxidative burst-mediated signalling in Arabidopsis. Nature 427: 858-861.
5. Clay NK, Adio AM, Denoux C, Jander G, Ausubel FM (2009) Glucosinolate metabolites required for an Arabidopsis innate immune response. Science 323: 95-101.
6. Colcombet J, Hirt H (2008) Arabidopsis MAPKs: a complex signalling network involved in multiple biological processes. The Biochemical Journal 413: 217-226.
7. Beckers GJ, Jaskiewicz M, Liu Y, Underwood WR, He SY, et al. (2009) Mitogen-activated protein kinases 3 and 6 are required for full priming of stress responses in Arabidopsis thaliana. The Plant Cell 21: 944-953.
8. Zentella R, Zhang ZL, Park M, Thomas SG, Endo A, et al. (2007) Global analysis of della direct targets in early gibberellin signaling in Arabidopsis. The Plant Cell 19: 3037-3057.
9. Czechowski T, Stitt M, Altmann T, Udvardi MK, Scheible WR (2005) Genome-wide identification and testing of superior reference genes for transcript normalization in Arabidopsis. Plant Physiology 139: 5-17.
10. Deshmukh S, Huckelhoven R, Schafer P, Imani J, Sharma M, et al. (2006) The root endophytic fungus Piriformospora indica requires host cell death for proliferation during mutualistic symbiosis with barley. Proceedings of the National Academy of Sciences of the United States of America 103: 18450-18457.
